# Risk factors for mortality in kidney transplant recipients with COVID‐19: a single centre experience and case–control study

**DOI:** 10.1186/s12882-022-02821-8

**Published:** 2022-07-07

**Authors:** Devprakash Choudhary, Deepesh Kenwar, Ajay Sharma, Ashish Bhalla, Sarbpreet Singh, Mini P Singh, Vivek Kumar, Ashish Sharma

**Affiliations:** 1 Department of Renal Transplant Surgery, Post Graduate Institute of Medical Education & Research (PGIMER), Chandigarh, 160012 India; 2 Royal Liverpool University Hospital, Liverpool, UK; 3 Department of Internal medicine, Post Graduate Institute of Medical Education & Research (PGIMER), Chandigarh, 160012 India; 4 Department of Virology, Post Graduate Institute of Medical Education & Research (PGIMER), Chandigarh, 160012 India; 5 Department of Nephrology, Post Graduate Institute of Medical Education & Research (PGIMER), Chandigarh, 160012 India

**Keywords:** COVID-19, Kidney Transplant Recipients, Co-infections, Diarrhoea, CMV

## Abstract

**Background:**

COVID-19 infection is considered to cause high mortality in kidney transplant recipients (KTR). Old age, comorbidities and acute kidney injury are known risk factors for increased mortality in KTR. Nevertheless, mortality rates have varied across different regions. Differences in age, comorbidities and varying standards of care across geographies may explain some variations. However, it is still unclear whether post-transplant duration, induction therapy, antirejection therapy and co-infections contribute to increased mortality in KTR with COVID-19. The present study assessed risk factors in a large cohort from India.

**Methods:**

A matched case–control study was performed to analyze risk factors for death in KTR (*N* = 218) diagnosed with COVID-19 between April 2020 to July 2021 at the study centre. Cases were KTR who died (non-survivors, *N* = 30), whereas those who survived were taken as controls (survivors, *N* = 188).

**Results:**

A high death-to-case ratio of 13.8% was observed amongst study group KTR infected with COVID-19. There was a high incidence (12.4%) of co-infections, with cytomegalovirus being the most common co-infection among non-survivors. Diarrhea, co-infection, high oxygen requirement, and need for mechanical ventilation were significantly associated with mortality on regression analyses. Antirejection therapy, lymphopenia and requirement for renal replacement therapy were associated with worse outcomes.

**Conclusions:**

The mortality was much higher in KTR who required mechanical ventilation and had co-infections. Mortality did not vary with the type of transplant, post-transplant duration and usage of depletion induction therapy. An aggressive approach has to be taken for an early diagnosis and therapeutic intervention of associated infections.

## Introduction

Coronavirus disease-19 (COVID-19), caused by severe acute respiratory syndrome coronavirus 2 (SARS-CoV-2), was declared a pandemic by the World Health Organization (WHO) in March 2020 [1]. COVID-19 infection in kidney transplant recipients (KTR) is known to cause high mortality compared to the general population, due to pre-existing comorbidities such as diabetes and hypertension [2, 3]. The mortality rate due to COVID-19 infection in KTR in a recent meta-analysis comprising of 4,440 patients ranged from 12 to 32% [2]. However, studies included in the meta-analysis were mainly from the developed nations like European countries and the USA and comprised mainly of elderly KTR with a mean age of survivors (mean ± SD) 54.9 ± 15.4 yrs vs non-survivors 67.5 ± 11.8 yrs respectively. There is limited published literature on KTR with COVID-19 infection from developing nations like India and Brazil, encompassing over 60% of COVID-19 cases globally [4].

The first confirmed case of COVID-19 in India was recorded on 27 January 2020 in Kerala. The first COVID-19 wave peaked in September 2020 (July-November 2020), and the second wave peaked in May 2021 (February – June 2021). By the end of the second COVID-19 wave in August 2021, India and Brazil stood at the second and third position respectively next to the USA, with a staggering over 31 million COVID-19 patients and 4,23,810 deaths alone in India [5]. Mortality rates of 11.6% and 21% amongst KTR have been reported from India and Brazil, respectively [6–8]. India rolled out COVID-19 vaccination in January 2021, and less than 20% of the Indian population had received both doses till the end of second-wave [5]. The vulnerability of KTR for COVID-19 infection remains despite vaccination because of ever-increasing mutant variants of SARS-CoV-2 [9, 10], The availability of newer monoclonal therapies, when given early, have been shown to reduce mortality [11]. Therefore, knowledge of risk factors contributing to increased mortality amongst KTR can lead to judicious use of these expensive and scarce resources. This retrospective study was performed using a case–control study design to analyze risk factors for death in KTR with COVID-19 infection from a developing nation perspective to improve quality of care and optimal resource utilization in a developing nation awaiting subsequent COVID-19 waves [12].

## Methods and materials

### Study design

This single centre retrospective matched case–control study was approved by the institutional ethics committee. A consent waiver was obtained from the ethics committee (No. INT/IEC/2021/NK/7997/Study/937).

### Study Population

218 KTR were diagnosed with COVID-19 infection from April 2020-July 2021. Cases (*n* = 30) were non-survivor KTR (*n* = 16, first wave and *n* = 14, second pandemic wave), and Controls (*n* = 188) were KTR survivors (*n* = 63, first wave and *n* = 125, second pandemic wave). To avoid the selection bias, up to 4 controls were individually matched from 188 surviving KTR to each case (non-survivor KTR) based on age(± 5 year), sex, pandemic wave period, and treatment status (hospital admission or home care-based treatment around the same time of evolving pandemic period). KTR who had received vaccination or experimental therapy (like hydroxychloroquine) or those with missing data and loss of follow-up were excluded.

### Diagnosis of COVID-19 and data collection

All KTR with COVID-19 were diagnosed by reverse transcriptase-polymerase chain reaction (RT-PCR) on nasopharyngeal swabs taken between April 2020-July 2021. KTR data were retrieved from the departmental electronic database, records from dedicated COVID-19 teleconsultations and face-to-face findings from the outpatient register. KTR were followed till 31 September 2021. Over 1400 patient months of follow-up were recorded amongst patients who survived the COVID-19 infection.

### *Variables* -Following parameters were compared between survivor vs non-survivor KTR

#### Baseline demographic and transplant-related characteristics of KTR with COVID-19

Age, sex, blood group, type of transplant (living donor vs deceased donor), post-transplant duration at the time of COVID-19 diagnosis, pre-existing comorbidities like diabetes, hypertension, maintenance immunosuppression, use of induction agent, history of delayed graft function(DGF), history of acute rejection and antirejection therapy in last three months before acquiring COVID-19 infection, history of tuberculosis/hepatitis B or C, recurrent COVID-19 infection, and administration of broad-spectrum antibiotics for recurrent bacterial infection in last three months before acquiring COVID-19 infection, ongoing graft dysfunction during COVID-19 infection, acute kidney injury as defined by KDIGO guidelines (increase in serum creatinine by 0.3 mg/dL or more within 48 h or increase in serum creatinine to 1.5 times baseline or more within the last seven days or urine output less than 0.5 mL/kg/h for 6 h) [13], allograft loss.

### COVID-19 symptoms and treatment-related

COVID-19 symptom spectrum, need for hospital admission, lymphopenia at the time of COVID-19 diagnosis, co-infections acquired during COVID-19 infection, mycophenolate mofetil (MMF) withdrawal or dose reduction, calcineurin inhibitors (CNI) withdrawal and steroid dose increment.

### COVID-19 management in KTR (Fig. 1)

**Fig.1:**
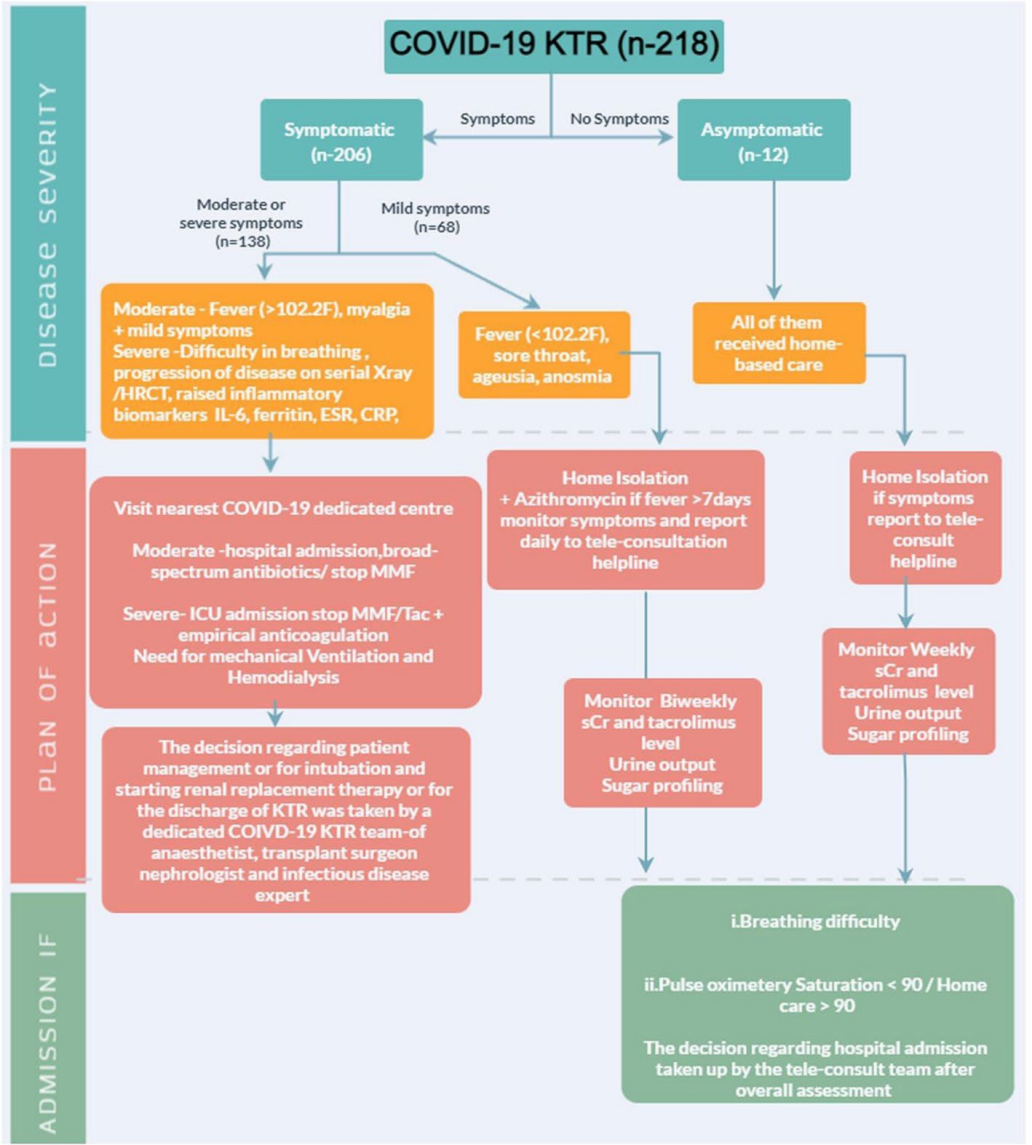
Covid-19 KTR (*n*-218)

The study centre is a public-funded tertiary institution providing care for all KTR diagnosed with COVID-19. In the first and second phases of the COVID-19 pandemic, KTR admitted with COVID-19 received dexamethasone 6 mg and remdesivir for five days in severe illness [14]. First drug to be withheld was antimetabolite, followed by tacrolimus, with an increment in steroid dose by 2.5 mg. The immunosuppressive drugs were recommenced gradually after a symptom-free period of one week, beginning with tacrolimus followed by half of the usual dose of antimetabolites for a week; as the recovery ensued, a full dose of antimetabolite was resumed.

### Statistical Analyses

The continuous variables were expressed as the median and interquartile range (IQR), and the categorical variables were expressed as counts and percentages. The normality of quantitative data was checked by measures of Kolmogorov Smirnov tests of normality. Fisher exact test or Chi-Square test was used to compare categorical variables. The student's t-test was used to compare the two independent groups in the normally distributed numerical data analyses and the Mann–Whitney-U test in the abnormal distribution of numerical data. Binary logistic regression analysis was used to assess independent parameters' relation with the primary outcome. *P* values of < 0.05 were considered significant. Variables that reached *P*-value ≤ 0.10 in the univariable analysis were selected for the regression analysis to determine the adjusted odds ratio. All analyses were performed by IBM SPSS 26 Statistics for Windows, Version 26 software.

## Results

The study population's death-to-case ratio was 30 out of 218 (13.8%).

### Baseline demographic and transplant-related characteristics of KTR with COVID-19 (Table 1).

**Table 1:** Baseline demographic, transplant-related characteristics, symptom and treatment-related factors of KTR with COVID-19, i.e., non-survivor vs survivor in KTR with COVID-19

Baseline Demographics and COVID Related Characteristics	Non-survivor (*n* = 30)	Survivor (*n* = 188)	*P*-value
	1st COVID Wave -*n* = 16 2nd COVID Wave- *n* = 14	1st COVID Wave -*n* = 63 2nd COVID Wave -*n* = 125	
Age *n* = (> 60(60–71) years:[median-64 yr] < 60 yr(14–60)[median -40 yr]	(8:22)	(15:173)	0.006
Sex (Male: Female)	23:7	146: 42	0.90
Blood Group (% within Blood Grp)			0.04
O	9 (30%)	39(20.74%)	
B	16 (53.33%)	71(37.76%)	
AB	3(10%)	19(10.11%)	
A	2 (6.66%)	59(31.38%)	
Living donor (n = 167) Vs deceased donor (n = 51)	21(70%) vs 9(30%)	146(77.65%) vs 42(22.34%)	0.39
Post-transplant duration Median (months) min–max	48.5(3–220)	54(0–234)	0.47
Interquartile range (25–50-75)	(28.25–48.5–97.25)	(24–54-86)	
Diabetes	12 (40%)	44 (23.40%)	0.05
Hypertension	28 (93.33%)	172(91.49%)	1
ATG induction	13 (43.33%)	107(56.91%)	0.17
Delayed graft function	2 (6.66%)	9(4.78%)	0.65
History of treatment for acute rejection in last three-month preceding COVID-19 infection	7 (23.33%)	20 (10.63%)	0.06
History of treatment for tuberculosis	4(13.33%)	22(11.70%)	0.76
Recurrent infection and antimicrobial therapy	7(23.33%)	34(18.08%)	0.46
History of hepatitis C infection	2(6.66%)	23(12.23%)	0.54
Baseline serum creatinine(mg/dl) Median min–max	1.45(0.8–7)	1.2(0.4–4.8)	0.002
Interquartile range	(1–1.45–1.85)	(1–1.2–1.4)	
Creatinine at presentation(mg/dl) Median Min–Max	3.05(0.76 -9.2)	1.3(0.3–7.5)	0.0001
Interquartile range	(1.80–3.05–4.12)	(1–1.3–1.7)	
Fever	26(86.66%)	160(85.11%)	1
Myalgia	20(66.66%)	130(69.15%)	0.83
Cough	16 (53.33%)	76 (40.42%)	0.23
Dyspnea	14 (46.66%)	48(25.53%)	0.02
Diarrhea	12 (40%)	28 (14.89%)	0.001
Loss of smell/ageusia	0	10(5.31%)	0.36
Community vs Hospital-acquired	28(93.33%) vs 2(6.66%)	181(96.28%) vs 7(3.72%)	0.35
Coinfection at time of COVID-19	9 (30%)	18(9.57%)	0.002
Lymphopenia at admission	13(43.33%)	31(16.49%)	0.001
CT CORAD^¥^ score of 4 or more	27(90%)	53(28.19%)	0.0001
Received antibiotics	28(93.33%)	139(63.76%)	0.019
Antimetabolite reduced or stopped	27(90%)	161(85.63%)	0.77
CNI stopped	23(76.66%)	38(20.21%)	0.001
Increase in steroids	14(46.66%)	53(28.19%)	0.38
On Going Graft dysfunction	20(66.66%)	35(18.61)	0.0001
Biopsy-proven acute rejection within three-month period of COVID-19 infection	7(23.33%)	10(5.31%)	0.003
Acute kidney Injury	17(56.66%)	29(15.42%)	0.0001
RRT® during COVID in KTR	13(43.33%)	11(5.85%)	0.0001
Oxygen requirements	30(100.0%)	45(23.93%)	0.0001
Intubation and mechanical ventilation	23(76.66.5%)	2(1.06%)	0.0001
Graft loss	3(10%)	7(3.72%)	0.14
Hospitalisation vs home care	27(90%) vs 3 (10%)	108(57.44%) vs 80(42.55%)	0.0001
Recurrent COVID (*n* = 2)	1(3.33%)	1(0.53%	0.25

¥ The coronavirus disease 2019 (COVID-19) Reporting and Data System (CO-RADS)

® RRT- Renal Replacement Therapy

The majority (89.4%) of the study population was less than 60 years of age, and 76.6% had received a kidney from a living donor. There was a preponderance of males in both groups (77.5%), which is the usual trend at our centre. KTR had comorbidities, with hypertension (91.7%) and diabetes (25.7%) being the most frequent. The primary cause of the end-stage renal disease (ESRD) in KTR was unknown in 52.3%, glomerulonephritis in 19.3%, diabetic nephropathy in 9.6%, autosomal dominant polycystic kidney disease (ADPKD) in 8.2%, lower urinary tract abnormalities in 3.4%, renal stone diseases in 3.2%, and others in 3.6%.

The median time from transplantation to COVID-19 infection was 48.5 (3 – 220) months for non-survivors and 54(0- 234)months amongst survivors. The mortality rate amongst patients with different post-transplant duration at the time of COVID-19 diagnosis was similar, i.e. till 1 year (13.8%), at 1–5 years (14.1%) and > 5 years (13.3%). (Table 2).

**Table 2:** Post-Transplant duration at the time of COVID diagnosis and its relation with outcomes

Duration	All KTR with COVID-19 (*n* = 218)	Death due to COVID-19 in KTR (*n* = 30)	Survivors	Risk of death
1 year	29 (13.30%)	4	25 (86.2%)	13.79%
1-5 year	99(45.41%)	14	85(85.8%)	14.1%
> 5 year	90(41.28%)	12	78(86.6%)	13.3%

### COVID-19 clinical presentation and Therapeutic intervention (*Table **1*)

Diarrhea was a more common feature among non-survivors (40%) than survivors (14.9%). 48.62% of the KTR (n-106) were initially managed on a home care basis, but 26 of these were subsequently hospitalized. Amongst the KTR (36.7%) managed only on a home care basis, no mortality was reported. Amongst the hospitalized KTR, 75/138 (54.3%) patients required intensive care support with high oxygen requirements, and 25 (32.5%) required mechanical ventilation. Thirty hospitalized patients (16 in the first and 14 in second wave) with COVID-19 died during the hospital stay. The median length of hospital stay among survivors was 8 (range 6–16) days; and 7 (range 2–14) days in non-survivors. Three KTR died at home after discharge from the hospital after a negative RT PCR report due to secondary infections (pulmonary tuberculosis and graft pyelonephritis) and inability to return to the study centre.

Among non-survivors, five were diagnosed with ongoing cytomegalovirus (CMV) infection on PCR, and two other KTR succumbed to pulmonary and rhino-orbital mucormycosis. Two KTR in the entire cohort developed recurrent COVID-19 after 122 and 135 days respectively, after two negative RT-PCR; one of them had a moderate illness during the first episode but developed a severe disease when reinfected with COVID-19 and succumbed to it, while the other survived both the infections [15].

The incidence of acute kidney injury (AKI) episodes and hemodialysis (HD) requirement was significantly higher in non-survivors KTR(*P *< 0.0001). Overall, 163 KTR had a rise in serum creatinine associated with COVID-19 infection. While most patients recovered their kidney function, creatinine did not return to baseline in 18/163 KTR. Additionally, 10 KTR lost their graft due to ongoing acute rejection (*n* = 5), non-adherence (*n* = 4) and recurrence of IgA nephropathy (*n* = 1) after recovery from their illness.

### Risk Factors Found to Be Associated with Mortality

To ascertain the confounding effect of variables, a logistic regression modelling test was performed by entering the significant variables associated with mortality in the univariate analysis, i.e. (age, blood group, diabetes, dyspnea, diarrhoea, coinfection at time of COVID-19, lymphopenia at admission, use of antibiotics, CNI withdrawal, pre-existing graft dysfunction, history of treatment for acute rejection or biopsy-proven acute rejection within three months of COVID-19 infection, acute kidney injury, RRT during COVID-19, hospitalization vs home care, Xray/CT changes, oxygen requirement, intubation and mechanical ventilation).

The test observed four significant predictors of mortality, i.e. need for intubation followed by high oxygen requirement, presence of co-infection, and diarrhea with odds ratio (CI limits) (Table 3) as per multinomial logistic regression analysis after adjusting for confounding factors. Results were estimated with Omnibus tests of model coefficients and showed adjusted estimate results.

**Table 3:** Multinomial Logistic regression

Transplant Related Characteristics			*P*- value	
Age			0.63	
Blood Group			0.09	
Diabetes			0.06	
History of treatment for acute rejection			0.81	
Baseline serum creatinine(mg/dl)			0.52	
Creatinine at presentation(mg/dl)			0.83	
Dyspnea			0.87	
**Diarrhoea**			0.027	
**Coinfection at time of COVID-19**			0.003	
Lymphopenia at admission			0.38	
Received antibiotics			0.25	
CNI stopped			0.39	
Ongoing Graft Dysfunction			0.83	
Biopsy-proven acute rejection with in three-month period of COVID-19 infection			0.56	
Acute kidney Injury			0.92	
Haemodialysis during COVID in KTR			0.89	
Hospitalisation vs home care			0.61	
Xray/Ct changes			0.93	
**Oxygen requirement**			0.003	
**Intubation and mechanical ventilation**			0.000	
Logistic regression adjusted Risk factor for Mortality in COVID-19				
	*P*-value	Odds Ratio	95% CI (lower)	95% CI (upper)
Diarrhoea	0.027	9.08	1.29	63.84
Coinfection at time of COVID-19	0.003	19.88	2.80	141.04
Oxygen requirement	0.003	36.62	3.51	381.63
Intubation and mechanical ventilation	0.000	326.08	31.36	3390.31

**Table 4:** Coinfections in Non-survivor’s vs Survivors

Co-Infection Among Non-survivor’s (*n* = 9)	Non-survivor’s (*n* = 30)		CO-Infection Among Survivors (*n* = 18)	Survivors (*n* = 188)	
	1^st^ COVID Wave (*n* = 16)	2^nd^ COVID Wave (*n* = 14)		1st COVID Wave (*n* = 14)	2nd COVID Wave (*n* = 125)
CMV (*n* = 5)	(*n* = 3)	(*n* = 2)	UTI (*n* = 11)	(*n* = 4)	(*n* = 7)
Mucormycosis (*n* = 2)	(n = 1)	(n = 1)	CMV(*n* = 3)		(*n*-3)
Pulmonary Tuberculosis	(*n* = 1)		BKV		(*n* = 1)
Graft Pyelonephritis	(*n* = 1)		Herpes Zoster		(*n* = 1)
			Hepatitis C		(*n* = 1)

On regression analysis, mortality did not vary with the type of transplant, post-transplant duration, usage of depletion induction, delayed graft function, history of treatment for previous tuberculosis or hepatitis C.

### Post Hoc Secondary Analysis: Association of CMV Coinfection a risk factor for Death During COVID-19 illness

The most common co-infection was CMV(16.7%) among non-survivors and urinary tract infections among survivors (5.8%) (Table 4). A total of eight CMV coinfection were diagnosed in the whole cohort of COVID-19 infected KTR; among them, five patients with CMV were diagnosed during the second COVID wave. Whereas all three KTRs with CMV infection died during the first wave, three out of five KTRs during the second wave survived as early recognition and timely antiviral therapy were instituted based on the earlier experience of the study centre.

### Sensitivity Analysis

Sensitivity analysis of excluding KTR and their matched controls who were not admitted to the hospital did not change the results.

## Discussion

COVID-19 has affected all aspects of a transplantation program, from donor selection to transplantation. The death to case ratio of 13.8% in KTR with COVID-19 infection at the study centre is similar (11.6%) to the reported multicentric study from India but much higher than the general Indian population (< 1.5%) [5, 6]. The reported mortality rate across the globe has been quite variable with a range of 12 to 32% amongst studies included in a recent meta-analysis of KTR [2]. The reasons for this wide variability are not clear. However, they might be related to differences in population demographics like the majority of KTR included in the meta-analysis were elderly, a higher prevalence of medical comorbidities like diabetes and quality of care received during the evolving pandemic. Among developing nations, KTR with COVID-19 infection has been reported to have a varying mortality rate of 9.5–11.6% in India and 21% in Brazil, possibly due to the higher median age of KTR in the Brazilian study group as compared current study group (51.3 vs 41 years, respectively) [7, 8]. Older age is a well-established risk factor for death among both KTR and the general population [16, 17].

The present study analyzed various risk factors contributing to mortality in KTR affected by COVID- 19 infection. Coinfection was an independent risk factor for mortality in the present study. This finding concords with a recent meta-analysis concerning coinfection with COVID-19, which showed a high prevalence (upto10%) of viral co-infections, which is associated with poor outcomes and further supports the need for diagnostic testing and treatment of associated infections [18]. In the present study group, cytomegalovirus infection was the commonest co-infection, and its presence has been associated with increased mortality amongst Iranian KTR with COVID-19 [19, 20]. CMV has a high prevalence (> 80%) in India amongst the adult population [21]. Reactivation of latent CMV infection occurs in > 30% of the seropositive patients receiving immunosuppression after kidney transplantation [22, 23]. Low absolute lymphocyte count is a known independent predictor of recurrent CMV disease in solid organ transplant recipients [24]. Lymphopenia at presentation among KTR with COVID-19 infection has been associated with increased mortality in the TANGO consortium [25]. It is possible that lymphocytopenia during severe SARS-CoV-2 infection might lead to cellular immune system deficiencies resulting in reactivation of CMV. CMV reactivation has also been reported in critically ill COVID-19 infected patients. CMV has been shown to increase the length of hospital stay among mechanically ventilated non-transplant patients in ICU and predispose them to other secondary infections [24, 26–28]. Routine screening of patients with severe or persistent lymphopenia for the presence of CMV infection might help in early diagnosis and timely intervention.

In the present study, monitoring of CMV in selected high-risk COVID-19 KTR helped us diagnose the disease early during the second wave. This strategy helped in the timely diagnosis and treatment of three KTR with CMV co-infection during the second COVID-19 wave. However, CMV testing was not done in the majority of the cases due to logistic issues during the lockdown when the medical and societal resources were overwhelmed. The clinical features of CMV like graft dysfunction, interstitial pneumonia with persistent lymphopenia, and diarrhoea are also present in COVID-19 infection.

The clinical spectrum of COVID-19 infection is broad. Therefore, it would be beneficial to understand any particular presentation with the risk of severe illness. Diarrhea at presentation was an independent risk factor for death amongst affected KTR in the present study and has been reported as a predictor for hospitalization but not for death among the Brazilian cohort of 1,680 KTR [8]. Several meta-analyses in patients with COVID-19 infection among non-transplant patients with gastrointestinal manifestations have reported that patients with diarrhoea were at much higher odds of increased disease severity and worse prognosis, and prolonged viral shedding in the GI tract has been shown in (> 20%-40%) of patients even after having a negative respiratory sample [29–33]. However, conflicting reports of diarrhea associated with lower mortality were observed in a meta-analysis of 4,440 KTR; the reason for this is not apparent [2]. Diarrhea in COVID-19 KTR has been proposed to result from direct infection of SARS-CoV-2 in the intestinal epithelium cells via angiotensin-converting enzyme 2 (ACE2) receptors, resulting in cytokine storm through direct cytopathic effects of SARS-CoV-2 contributing to gut dysbiosis and aberrant immune response resulting in increased intestinal permeability, which further exacerbates existing symptoms and worsen prognosis [34, 35]. Gastrointestinal SARS-CoV-2 infection has important epidemiological significance as diarrheal diseases are more common in developing nations, and SARS-CoV-2 in the faeces may facilitate the spread of COVID-19 through the faecal-oral transmission and contamination of surroundings [36]. Gut-lung axis and GI dysbiosis in COVID-19 have been postulated as contributory factors for the severity of SARS-CoV-2 illness [37, 38]. Due to limited healthcare resources, the present study group did not test for faecal samples among KTR with COVID-19 infection.

No differences in patient survival were found depending on the time after kidney transplantation among 138 hospitalized KTR with COVID-19, suggesting that continuing transplant activity may not put transplant patients in the early postop period with additional risk [39]. In contrast, higher mortality in the early post-transplant period (< 60 days) was observed among KTR from Spain in the early phase of the pandemic conceivably due to the inclusion of elderly KTR (> 60 years) [40]. Modification in induction immunosuppression regimens was performed during the COVID-19 pandemic at many centres, with some initial success in kidney transplantation, while the standard ATG induction therapy was associated with the risk of severe COVID-19 illness [41–43]. The study centre used a low dose induction therapy with doses up to 3 mg/kg, and this was not associated with increased mortality among KTR during the COVID-19 pandemic. A similar finding was observed in a retrospective study from Poland regarding the safe usage of standard dosage of ATG in KTR both as induction and antirejection therapy during the COVID-19 pandemic [44]. However, antirejection therapy during the last three months before acquiring COVID-19 infection was associated with worse outcomes on univariate analysis in the present study, in agreement with a multicentric study from India [6]. Ongoing graft rejection during the COVID-19 infection was another risk factor for death among KTRs on univariate analysis, possibly related to the intensification of immunosuppression in the presence of graft dysfunction.

Furthermore, Calcineurin inhibitor (CNI) withdrawal during the hospital stay was a risk factor for mortality on univariate analysis in the study cohort, and continued use of tacrolimus has been associated with better survival amongst liver transplant recipients requiring hospitalization in the ELITA/ELTR multicentre European study [45]. Withdrawal of tacrolimus is an integral part of managing sick KTR, and the benefits of continuing it in KTR with severe COVID-19 infection need to be confirmed in future studies as patients with COVID-19 infection have also been associated with cytokine storm during the immuno-inflammatory phase that is more likely to happen if the immunosuppression is suddenly lowered [46].

COVID-19 related mortality in this study is also attributed to the difficulty accessing healthcare facilities during the pandemic. The study centre caters to patients with limited health care resources, thereby attracting transplant recipients from far-flung regions. The lockdowns resulted in the discontinuation of routine outpatient services. Markedly reduced public transport options made it further difficult for those patients who cannot afford private health care facilities [47].

Our data does not show any influence of hypertension as comorbidity, type of transplantation (living versus deceased donor) and delayed graft function on mortality figures mainly due to the younger age group and typically shorter cold ischemia time in recipients of deceased donor kidneys at the study centre, but this could be a type 2 error because of too much scattering of data or inadequate sample size. Inferior allograft function among the deceased donor KTR than living donors KTR has been associated with an increased risk of severe COVID-19 infection in a recent meta-analysis [2].

Other well-established risk factors for mortality in KTR due to COVID-19 infection were also significant in univariate analysis in the present study, i.e. elderly KTR, diabetes as comorbidity, dyspnea, acute kidney injury, lymphopenia, the requirement of renal replacement therapy and are in accord with already published literature [2, 3, 6, 8, 16, 25].

### Limitations of study

Limited laboratory tests for inflammatory biomarkers (interleukin-6, C-reactive protein) have been available due to logistic issues during the pandemic. This study presumes that the distribution of the risk of exposure was the same in both groups, i.e. survivors vs non-survivors. In addition, this study presumes that the promptness and economic factors that play a role in access to health care, especially when the health care systems are overstretched during the pandemic, were similar in both groups. It is impossible to eliminate or mitigate against three types of biases in a retrospective study, i.e., selection, recall, and observer bias.

### Strengths of study

The study is endowed with many positive features such as matched study design with extensive workup, regular and comprehensive follow-up, a robust data extraction facility and a finely monitored data collection process that is regularly cross-checked by a dedicated team of residents in-charge of COVID-19 team who ensured that missing data was kept to a minimum. The quality of information is reliable and reproducible.

## Conclusions

COVID-19 infection was associated with high mortality in KTR, which significantly increased in patients with co-infections or diarrhea as presenting feature. Routine screening for co-infections like CMV might further reduce mortality in high-risk group KTR.

## Disclosures

There are no financial conflicts of interest to disclose. There is no conflict of interest of any of the authors regarding its publication.

## Data Availability

Raw data were generated at the Department of Renal Transplant Surgery PGIMER Chandigarh. The data that support the findings of this study are available on reasonable request from the corresponding author. The data are not publicly available as consent waiver is obtained.

## Abbreviations

AKI: Acute kidney injury
ARDS: Acute respiratory distress syndrome
CFR: Case fatality rate
CMV: Cytomegalovirus
CNI: Calcineurin inhibitor
HRCT: High-resolution computerized tomography
KTR: Kidney transplant recipients
MMF: Mycophenolate mofetil
RRT: Renal Replacement Therapy
RT-PCR: Reverse transcriptase-polymerase chain reaction
SCR: Serum creatinine
